# Efficacy Analysis of Evidence-Based Nursing Combined With Computer-Assisted Cognitive Correction Therapy on Cognitive Function and Quality of Life in Patients With Schizophrenia

**DOI:** 10.62641/aep.v54i4.2325

**Published:** 2026-08-15

**Authors:** Yinai Zhao, Xiulong Liu

**Affiliations:** ^1^Department of Nursing, Qingtian Kangning Hospital, 323900 Lishui, Zhejiang, China; ^2^Department of General Psychiatry, Qingtian Kangning Hospital, 323900 Lishui, Zhejiang, China

**Keywords:** evidence-based nursing, computer-assisted cognitive remediation, schizophrenia, cognitive function, quality of life

## Abstract

**Background:**

To investigate the effects of evidence-based nursing combined with computer-assisted cognitive remediation on cognitive function and quality of life in patients with schizophrenia.

**Methods:**

A retrospective analysis was conducted on the clinical data of 120 patients with schizophrenia admitted between March 2023 and March 2025. Patients were divided into an observation group (n = 60, receiving evidence-based nursing combined with computer-assisted cognitive remediation) and a control group (n = 60, receiving routine nursing combined with routine cognitive training) according to their treatment and nursing protocols. Continuous treatment included in-hospital and post-discharge 12 weeks. Cognitive function, including the Wisconsin Card Sorting Test (WCST), Digit Symbol Substitution Test (DSST), Semantic Verbal Fluency (sVF) task, and the Schizophrenia Patient Quality of Life Scale (SQLS), was compared between the two groups before and 12 weeks after treatment.

**Results:**

Before treatment, there were no statistically significant differences in the scores of WCST, DSST, sVF, and SQLS dimensions between the two groups (*p* > 0.05). After 12 weeks of treatment, all cognitive indices showed significant within-group improvements for both groups (all *p* < 0.001), while each SQLS dimension and total score were significantly reduced within each group (observation group: all *p* < 0.01; control group: all *p* < 0.05). After Bonferroni correction for multiple comparisons, all cognitive and SQLS outcomes showed significantly larger improvements in the observation group relative to the control group (all corrected between-group *p* < 0.001).

**Conclusions:**

Evidence-based nursing combined with computer-assisted cognitive correction therapy is associated with better cognitive performance and higher quality of life in patients with schizophrenia, showing promising clinical application value.

## Introduction

Schizophrenia is a serious, chronic, and relapsing mental illness that is prevalent worldwide. Patients have typical positive symptoms such as hallucinations and delusions, as well as negative symptoms such as emotional apathy, lack of will, and social withdrawal. It has been listed by the World Health Organization as one of the major public health problems [1]. Cognitive impairment is also one of the core symptoms of schizophrenia, and this impairment runs through the entire course of the disease. Even before the appearance of positive symptoms, there may be hidden damage [2]. Clinical studies have shown that schizophrenia patients have varying degrees of cognitive impairment, mainly manifested as multiple-dimensional defects such as inattention, working memory loss, executive dysfunction, slowed information processing speed, and decreased verbal fluency [3]. Moreover, cognitive impairment has a more lasting and profound impact on patients. It directly hinders patients’ correct perception and judgment of the surrounding environment, weakens their ability to learn new knowledge and master new skills, and thus seriously affects patients’ social interaction, career return, and daily life self-care ability [4, 5, 6].

Cognitive impairment is not only a key factor leading to a decline in the quality of life for patients with schizophrenia, but also places a heavy burden on families and society. Related studies show that the quality of life scores of patients with schizophrenia are significantly lower than those of the general population, with a positive correlation between the degree of cognitive impairment and the degree of decline in quality of life [7, 8]. For patients’ families, the long-term care needs not only consume a significant amount of time, energy, and financial resources, but caregivers themselves are also prone to psychological problems such as anxiety and depression [9, 10]. From a societal perspective, the loss or decline in the ability to work of patients with schizophrenia, coupled with disease-related medical expenses and social assistance expenditures, imposes a huge economic burden on society every year [11, 12]. Therefore, improving the cognitive function of patients with schizophrenia, thereby enhancing their quality of life, is not only a necessity to protect the individual health rights of patients, but also an important measure to reduce the burden on families and society and maintain social harmony and stability.

Currently, the clinical treatment of schizophrenia still mainly relies on antipsychotic drugs, which are also the primary means of controlling patients’ positive symptoms. However, while antipsychotic drugs can effectively relieve acute symptoms such as hallucinations and delusions and reduce the risk of disease relapse, their effect on improving cognitive function is very limited [13, 14]. It is difficult to achieve comprehensive recovery for patients with schizophrenia by relying solely on drug treatment. Finding safe, effective, and feasible non-drug treatments to work synergistically with drug treatment to improve patients’ cognitive function and enhance their quality of life has become a key research focus and hot topic in the field of clinical nursing and rehabilitation in psychiatry.

In the care of patients with schizophrenia, evidence-based nursing can focus on the core issues of patients’ cognitive function deficits, psychological state, and self-care ability. It can search for the latest research evidence at home and abroad, and combine the patient’s specific condition, personality characteristics and family environment to formulate a comprehensive nursing plan including cognitive treatment, psychological counseling, life skills training and family support guidance, so as to help patients recover in all aspects. Computer cognitive correction therapy is a systematic and modular cognitive training method developed based on the theory of cognitive neuroscience. It constructs diverse training scenarios through computer programs and conducts precise and step-by-step training for specific cognitive deficit dimensions such as patients’ attention, memory and executive function [15]. Compared with traditional cognitive training, computer cognitive correction therapy has significant advantages such as standardized training content, quantifiable training process, high patient participation and convenient operation. It can adjust the training difficulty in real time according to the patient’s training progress and effect, ensuring the scientificity and effectiveness of training [16].

While both evidence-based nursing and computer-assisted cognitive remediation therapy (CCRBT) have demonstrated positive effects in the rehabilitation of patients with schizophrenia, research on the synergistic efficacy of their combined application remains incomplete. Therefore, this study retrospectively analyzed the clinical data of 120 patients with schizophrenia to explore the impact of evidence-based nursing combined with CCRBT on patients’ cognitive function and quality of life. It aims to provide certain objective data and decision-making basis for optimizing rehabilitation strategies for schizophrenia patients, helping more patients improve their cognitive function, enhance their quality of life, and achieve better social reintegration.

## Materials and Methods

### Research Subjects

A total of 179 hospitalized schizophrenia patients were initially screened for eligibility at Qingtian Kangning Hospital from March 2023 to March 2025. Inclusion criteria: (1) meeting the diagnostic criteria for schizophrenia in the Diagnostic and Statistical Manual of Mental Disorders (5th Edition) (DSM-5) [17]; (2) aged 18 to 60 years, regardless of gender; (3) disease duration ≥ 6 months, with clear cognitive impairment; (4) During hospitalization, patients received standardized antipsychotic medication, and clinical data and treatment records were complete and traceable; Exclusion criteria: (1) Medical records record the existence of serious organic diseases of important organs such as heart, liver, and kidney; (2) Medical records confirm diagnosis of organic brain disease, intellectual disability, alcohol or drug dependence; (3) Medical records record serious suicidal tendencies, aggressive behavior, or inability to cooperate in completing cognitive function assessment; (4) Were pregnant or lactating during treatment; (5) Participated in other cognitive treatment clinical studies within 3 months of treatment.

Of these candidates, 59 subjects were excluded for the following reasons: 17 patients complicated with severe organic visceral diseases or organic brain lesions, 12 cases with alcohol or psychoactive substance dependence, 19 patients unable to finish complete cognitive assessment owing to severe aggressive or suicidal behaviors, 7 pregnant/lactating women, and 4 participants enrolled in other cognitive treatment trials within 3 months. Finally, 120 eligible patients were included and allocated into the observation group (n = 60) and control group (n = 60) based on actual implemented nursing and rehabilitation protocols. This study was reviewed and approved by the Medical Ethics Committee of Qingtian Kangning Hospital (ethics approval number: X-2026-01-19). All procedures performed in this study involving human participants were in accordance with the ethical standards laid down in the Declaration of Helsinki. The research will be carried out in strict accordance with the version of the protocol that has received ethical approval. Written informed consent was obtained from all schizophrenia patients as well as their respective legal guardians.

### Methods

The clinical data of both groups of patients extracted from the medical record system showed that all patients received routine antipsychotic medication treatment, with the type and dosage adjusted by clinicians according to the patients’ conditions, and the medication information was completely recorded in the medical record system. According to the actual nursing and cognitive treatment measures received by patients during hospitalization and recorded in the medical record system, the patients were divided into two groups: the control group received routine nursing care combined with routine cognitive training, and the observation group received evidence-based nursing care combined with computer-assisted cognitive correction therapy. The total treatment cycle for both groups was 12 weeks, with the first 4 weeks delivered during hospitalization and the remaining 8 weeks continued via standardized post-discharge follow-up and home-based treatment guidance; and all treatment implementation records, including frequency, duration and content, were completely preserved in the medical record system.

#### Control Group

The clinical data showed that the routine nursing care and routine cognitive training received by the control group during hospitalization were the conventional clinical measures of our hospital, and the specific implementation content was completely recorded in the nursing records and medical records: (1) Routine nursing care: Vital sign monitoring was performed twice daily (8:00 AM & 8:00 PM), with abnormal values reported immediately; a unified low-salt and low-fat diet was provided three times a day, with food intake recorded and treatment for insufficient intake. Sleep duration was tracked daily (≥6 h nighttime sleep required), and medication was dispensed and supervised to ensure adherence. The ward was cleaned twice daily, ventilated for 30 min twice daily and disinfected weekly with chlorine-containing disinfectant. Hazard inspections were conducted three times daily, with routine patients patrolled every 30 min and high-risk patients every 15 min. Group health education was held twice weekly for 20 min (fixed content: disease knowledge, drug adverse reactions, nursing precautions), with core knowledge awareness rate ≥80% verified by oral questioning. (2) Routine cognitive training: It is conducted by trained nurses using unified and customized tools (number/Chinese characters/arithmetic/graphic reasoning cards, number elimination cards). Attention training (5 min to eliminate numbers between 1–50), memory training (10 sets of 3–5 digits/characters, 10s display) and logical thinking training (20 arithmetic questions/10 graphic reasoning questions, time limit 10 min), the basic difficulty is fixed and will not be gradually upgraded; the completion accuracy and time are recorded immediately after training. According to the nursing operation records, the training is held 3 times a week, 30 minutes each time (5 min of explanation + 20 min of core training + 5 min of summary), and the time is 3:00 PM during hospitalization; after discharge, patients completed the same training content 3 times a week under family supervision, with remote follow-up checks by nurses every week. All nursing operations and training details are fully recorded, with a record completion rate of 100%.

#### Observation Group

Clinical data show that the observation group received evidence-based nursing combined with computer-assisted cognitive correction therapy during their hospitalization. In addition, they received the same basic routine nursing care as the control group (e.g., vital sign monitoring, diet and sleep management, medication supervision). The specific implementation process and details were fully recorded in specialist nursing records, treatment records and medical records: (1) Evidence-Based Nursing: (a) The evidence-based nursing team of our hospital, consisted of 1 associate chief nurse, 2 head nurses, and 3 nurses with relevant evidence-based nursing training and rich clinical nursing experience in psychiatry, was responsible for the implementation of nursing measures. (b) The team formulated evidence-based nursing plans for schizophrenia patients based on the clinical core issues of improving cognitive function and quality of life, and the plan formulation process was based on the latest research evidence at home and abroad and combined with clinical experience. (c) The personalized evidence-based nursing plan implemented for the patients included four aspects, with standardized core content and individualized adjustments based on each patient’s cognitive deficits, emotional state, and family environment. Cognitive nursing treatment: The nurse guided patients to carry out targeted attention, memory and executive function auxiliary training, including simple number memory, classification sorting, daily problem simulation and other operations; the training type, duration, and basic difficulty are fixed according to standards, but the difficulty and task selection are personalized based on baseline cognitive performance; cooperate with computer cognitive training. Psychological nursing treatment: One-to-one emotional communication and relaxation guidance were carried out to relieve anxiety, low mood and training resistance, and encourage patients to adhere to rehabilitation; the communication framework and relaxation techniques are implemented according to unified standards; however, coping strategies and frequency of communication that vary in emotional severity and treatment compliance must be individualized for each situation. Social function rehabilitation nursing: Simulated daily communication, ward collective interaction and simple social scene training were carried out to improve expression and communication ability, social scenes and interaction protocols are fixed and unified; however, patients with different scene complexity and social skill levels need to find matching interaction partners. Family support nursing: The contents of disease knowledge, training cooperation and home supervision were explained to family members, and a short family communication guidance was conducted; the teaching materials and core guidance themes remain the same; but the depth of guidance and family treatment plan are personalized according to the family’s understanding and care ability. The whole process was recorded in the nursing record, the core procedures and basic content are standardized and fixed, ensuring consistency and repeatability, while the specific implementation details are personalized to meet the unique needs of each patient. According to the nursing operation records, the evidence-based nursing treatments was conducted 2 times a week for 20 minutes each time during the hospitalization; after discharge, weekly telephone follow-up and family guidance were provided to sustain the nursing effect for the remaining treatment period. (2) Computer-Assisted Cognitive Therapy: The patients received training through the Computer-Assisted Cognitive Therapy System (VCRT-G, Nanjing Weisi Medical Technology Co., Ltd.) of our hospital. This system includes multiple training modules such as attention, memory, executive function, and information processing speed. Before the implementation of the training, professional medical staff conducted a systematic assessment of the patients and formulated personalized training plans, with the assessment results and plan content recorded in the medical records. During the training, nursing staff provided on-site guidance, the patients completed the designated training tasks through the computer, and the system automatically recorded the training progress and effects, with the training difficulty adjusted every two weeks based on the actual training results. According to the treatment operation records, the computer-assisted cognitive therapy was conducted 3 times a week for 30 minutes each time during hospitalization via the hospital’s system; after discharge, patients used a simplified home-based computer training module to complete 3 sessions per week, with training data synchronized to the hospital system for real-time supervision.

### Observation Indicators

All observation indicators in this study were extracted and sorted from the hospital medical record system, including the clinical baseline data of both groups of patients (gender, age, disease duration, education level, type and dosage of medications, Positive and Negative Syndrome Scale (PANSS) scores including total score and negative symptom subscore, illness phase and prior rehabilitation exposure) recorded in the medical records, and the cognitive function and quality of life assessment results of patients before and 12 weeks after the implementation of treatment measures. The cognitive function and quality of life assessment were completed by independent, trained professional assessors who were blinded to group allocation of the patients. All assessors received uniform professional training, and the assessment process strictly followed the standardized operating procedures, with the original assessment results completely recorded in the medical record system.

#### Cognitive Function Assessment

Cognitive assessment covered multiple dimensions, all administered by blinded assessors unaware of group assignment, including: (1) Wisconsin Card Sorting Test (WCST) [18]: mainly assesses the patient’s executive function, and the recorded indicators include the total number of correct answers, the total number of errors, and the number of persistent errors; all participants finished a fixed total of 128 test responses in both pre-treatment and post-treatment WCST assessments; the total response quantity was identical among all subjects and consistent at two assessment time points. Perseverative errors were defined as repeated incorrect responses continuously adhering to an abandoned sorting rule, whereby consecutive responses following an invalid classification criterion were counted as perseverative errors instead of isolated single wrong answers. (2) Digit Symbol Substitution Test (DSST) [19]: mainly assesses the patient’s information processing speed and attention, and the recorded indicator is the number of correct digit symbol matches completed; the test sheet contained a fixed total of 90 digit-symbol matching items; all participants were required to complete matching tasks sequentially within a unified time limit of 90 to 120 seconds, and the identical test form, total item quantity and time restriction were applied for both pre-treatment and post-treatment assessments across all subjects. (3) Semantic Verbal Fluency (sVF) task [20]: mainly assesses the patient’s verbal expression ability and semantic memory, and the recorded indicator is the number of words of a specified category (such as animal or fruit) spoken by the patient within 1 minute.

#### Quality of Life Assessment

The Schizophrenia Patient Quality of Life Scale (SQLS) [21] was used by blinded professional assessors unaware of group allocation to assess the quality of life of patients during clinical treatment, and the assessment results were recorded in the medical record system. This scale comprises three dimensions: psychosocial functioning (15 items), energy/motivation (7 items), and symptoms/side effects (8 items), with a total of 30 items. All individual items adopt a 5-point Likert scoring from 0 (never) to 4 (always). According to the SQLS scoring manual, four positively worded items in the energy/motivation subscale were reversely recoded before raw score calculation using the transformation: original score 4→0, 3→1, 2→2, 1→3, 0→4. Each subscale’s raw total score was converted to a standardized domain score ranging from 0 (best health) to 100 (worst health) using the formula: Domain standardized score = (total raw score of subscale/maximum possible raw score of this subscale) × 100. The overall SQLS total score was calculated as the arithmetic mean of the three standardized subscale scores, also ranging 0–100. Higher scores indicate a poorer quality of life. Scores for each dimension and the total score were calculated by the assessors and recorded in the medical record system to reflect the patients’ overall quality of life.

### Data Processing

SPSS 26.0 (IBM Corp., Armonk, NY, USA) statistical software was used for data processing and analysis of the research data extracted from the medical record system. Normally distributed continuous data were expressed as Mean ± standard deviation ($\bar{x}$ ± s). All hypothesis tests in this study adopted two-sided testing, with α = 0.05 as the statistical threshold. For primary outcomes, a two-way repeated-measures analysis of variance (RM-ANOVA) was applied, with group as the between-subjects factor and time as the within-subjects factor; the primary test focused on the main effect of group, main effect of time, and group-by-time interaction effect. To control type I error from multiple comparisons across cognitive and quality of life outcomes, Bonferroni correction was applied for post-hoc between-group comparisons. Paired *t*-tests were used for within-group comparisons before and after treatment, and independent samples *t*-tests were used for post-treatment between-group comparisons. Count data were expressed as percentages (%), and inter-group comparisons were performed using the χ^2^ test. A *p*-value < 0.05 was considered statistically significant.

## Results

### Comparison of Baseline Clinical Data Between the Two Groups of Patients

Baseline data demographics, disease duration, education, medication, symptom severity (PANSS total score), negative symptoms (PANSS negative subscore), illness phase, and prior rehabilitation exposure were retrospectively collected for both groups. Statistical analysis showed that there were no statistically significant differences in the above baseline data between the two groups (*p* > 0.05), indicating comparability (see Table 1).

**Table 1.** **Comparison of baseline clinical data between the two groups of patients**.

Index		Observation group (n = 60)	Control group (n = 60)	*t*/χ^2^	*p*
Gender (n, %)				0.133	0.715
	Female	28 (46.67)	30 (50.00)		
	Male	32 (53.33)	30 (50.00)		
Age (years, $\bar{x}$ ± s)		38.62 ± 8.35	39.25 ± 7.98	0.396	0.693
Disease duration (years, $\bar{x}$ ± s)		5.31 ± 2.14	5.58 ± 2.36	0.687	0.493
Education level (n , %)				0.372	0.830
	Primary school and below	10 (16.67)	12 (20.00)		
	Junior high school	25 (41.67)	23 (38.33)		
	High school and above	25 (41.67)	25 (41.67)		
Types of medications (n , %)				0.258	0.878
	Risperidone	22 (36.67)	24 (40.00)		
	Olanzapine	20 (33.33)	19 (31.67)		
	Quetiapine	18 (30.00)	17 (28.33)		
Risperidone dosage (mg/day, $\bar{x}$ ± s)		3.52 ± 0.86	3.65 ± 0.92	0.725	0.470
	Olanzapine dosage (mg/day, $\bar{x}$ ± s)	14.28 ± 2.56	14.52 ± 2.63	0.458	0.647
	Quetiapine dosage (mg/day, $\bar{x}$ ± s)	526.35 ± 89.42	531.68 ± 92.15	0.326	0.745
	PANSS total score ($\bar{x}$ ± s)	75.28 ± 10.36	76.14 ± 9.87	0.451	0.653
	PANSS negative subscore ($\bar{x}$ ± s)	22.15 ± 4.28	22.87 ± 4.51	0.892	0.374
Illness phase (n, %)				0.315	0.854
	Acute	18 (30.00)	16 (26.67)		
	Stable	42 (70.00)	44 (73.33)		
Prior rehabilitation exposure (n, %)				0.148	0.701
	Yes	25 (41.67)	13 (21.67)		
	No	35 (58.33)	47 (78.33)		

Note: PANSS, Positive and Negative Syndrome Scale.

### Comparison of Cognitive Function Scores Between the Two Groups of Patients

Before the treatment, there were no statistically significant differences in the scores of the WCST, DSST and sVF between the two groups (*p* > 0.05). RM-ANOVA revealed significant main effect of group, main effect of time, and group-by-time interaction effects for all cognitive indices (all *p* < 0.001), indicating that cognitive function improved significantly over time overall, there were overall inter-group differences in cognitive performance, and the magnitude of pre-post cognitive improvement differed substantially between groups. After 12 weeks of treatment, paired t-tests showed significant within-group cognitive improvements in both groups (all *p* < 0.001). Post-hoc intergroup comparisons adjusted by Bonferroni correction to control type I error from multiple cognitive outcome tests further demonstrated that post-treatment cognitive scores were significantly better in the observation group relative to the control group (all *p* < 0.001). See Table 2.

**Table 2.** **Cognitive function scores: within-group pre-post changes and between-group post-treatment comparisons ($\bar{x}$ ± s)**.

Indicator		WCST			DSST	sVF
		Correct answers	Errors	Persistent errors	Correct matches	Word count
Observation group (n = 60)	Pre-treatment	32.15 ± 5.32	28.63 ± 6.25	18.32 ± 4.56	25.36 ± 4.87	12.58 ± 3.24
	Post-treatment	48.62 ± 6.18*	12.35 ± 4.18*	6.15 ± 2.38*	42.85 ± 5.63*	26.34 ± 4.15*
	Within-group *t*	15.236	16.982	18.543	17.321	19.654
	Within-group *p*	<0.001	<0.001	<0.001	<0.001	<0.001
Control group (n = 60)	Pre-treatment	31.87 ± 5.46	29.15 ± 6.31	18.67 ± 4.62	24.98 ± 4.92	12.36 ± 3.31
	Post-treatment	39.54 ± 5.83	20.46 ± 4.52	12.58 ± 3.15	33.67 ± 5.24	18.75 ± 3.86
	Within-group *t*	8.345	7.896	8.124	8.675	9.234
	Within-group *p*	<0.001	<0.001	<0.001	<0.001	<0.001
RM-ANOVA Main Effects and Interaction	Main effect of time (*F*)	216.352	241.781	268.945	235.671	292.458
	Main effect of time (*p*)	<0.001	<0.001	<0.001	<0.001	<0.001
	Main effect of group (*F*)	48.691	53.274	61.358	57.842	72.169
	Main effect of group (*p*)	<0.001	<0.001	<0.001	<0.001	<0.001
	Group × time interaction (*F*)	12.673	14.285	16.932	13.854	15.721
	Group × time interaction (*p*)	<0.001	<0.001	<0.001	<0.001	<0.001

Note: WCST, Wisconsin Card Sorting Test; DSST, Digit Symbol Substitution Test; sVF, Semantic Verbal Fluency; RM-ANOVA, repeated-measures analysis of variance. *p < 0.001 vs. control group (post-treatment score, independent samples *t*-test).

### Comparison of Quality of Life Scores Between the Two Groups of Patients

Before the treatment, there were no statistically significant differences in the scores of each dimension and the total score of the SQLS between the two groups (*p* > 0.05). RM-ANOVA showed significant main effect of group, main effect of time, and group-by-time interactions for all SQLS outcomes (all *p* < 0.001). The results suggested that quality of life improved significantly over time in all patients, overall quality of life differed between the two groups, and the degree of decline in SQLS scores differed across groups over the treatment period. After 12 weeks of treatment, paired *t*-tests demonstrated significantly reduced SQLS scores in both groups: within-group *p* values were all <0.01 for the observation group, and ranged from 0.017 to 0.029 for the control group (all *p* < 0.05). Post-hoc intergroup comparisons adjusted via Bonferroni correction to mitigate inflation of type I error across multiple SQLS subscale and total score outcomes confirmed that all post-treatment SQLS dimension scores and total score were significantly lower in the observation group compared with the control group (All *p* < 0.001), see Table 3.

**Table 3.** **Comparison of SQLS scores before and after treatment between the two groups of patients ($\bar{x}$ ± s)**.

Index	Time point	Psychosocial function	Energy/Motivation	Symptoms/Side effects	SQLS total score
Observation group (n = 60)	Pre-treatment	57.84 ± 7.63	54.17 ± 6.75	51.63 ± 6.29	54.91 ± 6.72
	Post-treatment	46.12 ± 6.41△	42.35 ± 5.28△	38.02 ± 4.76△	41.74 ± 5.39△
	Within-group *t*	8.761	9.324	9.953	10.869
	Within-group *p*	0.004	0.003	0.003	0.002
Control group (n = 60)	Pre-treatment	58.11 ± 7.49	54.42 ± 6.88	51.28 ± 6.35	54.66 ± 6.81
	Post-treatment	51.76 ± 6.83	47.69 ± 5.77	43.15 ± 5.19	47.38 ± 5.82
	Within-group* t*	4.314	4.767	5.433	5.891
	Within-group *p*	0.029	0.024	0.020	0.017
RM-ANOVA Main Effects and Interaction	Main effect of time (*F*)	189.462	207.835	224.157	213.694
	Main effect of time (*p*)	<0.001	<0.001	<0.001	<0.001
	Main effect of group (*F*)	42.375	49.681	56.243	53.716
	Main effect of group (*p*)	<0.001	<0.001	<0.001	<0.001
	Group × time interaction (*F*)	10.241	11.568	13.072	12.159
	Group × time interaction (*p*)	<0.001	<0.001	<0.001	<0.001

Note: SQLS, Schizophrenia Patient Quality of Life Scale; RM-ANOVA, repeated-measures analysis of variance. △ Post-treatment score was significantly lower than that of the control group.

## Discussion

Cognitive impairment in patients with schizophrenia is a key and difficult point in clinical rehabilitation treatment. It not only affects the control of patients’ symptoms, but also directly determines their quality of life and ability to reintegrate into society [22]. This study retrospectively analyzed the clinical data of 120 patients with schizophrenia to explore associations between combined treatment and patients’ cognitive function and quality of life. The results indicated that the combined treatment was correlated with better cognitive level and superior quality of life.

In addition to behavioral improvements, the superior outcomes associated with combination treatment may be related to neuroplastic changes within cortical networks. Computer cognitive correction therapy provides repeated progressively challenging exercises to drive experience-dependent neuroplasticity. The studies have shown that this training can enhance activation of the prefrontal cortex and improve functional connectivity between prefrontal regions and areas critical for executive function and attention [23, 24]. From the perspective of neuropsychological rehabilitation theory, the synergistic effect of evidence-based care lies in optimizing the conditions for neuroplasticity: psychological care reduces emotional distress and motivational deficits, and reduces cognitive load; family support and social rehabilitation provide a rich environment that can strengthen newly acquired cognitive strategies [25, 26]. This integration of cognitive remediation with emotional and social scaffolding helps generalize training outcomes to real-world functioning, translating changes at the neural level into improved cognitive performance and quality of life.

Consistent with the repeated-measures ANOVA results, both groups demonstrated significant cognitive improvements after 12 weeks, while the observation group showed substantially greater gains. The significant group-by-time interactions confirm that the combined treatment yielded superior effects relative to routine care, supporting the synergistic benefit of evidence-based nursing plus computer-assisted cognitive remediation. Computer cognitive correction therapy, as a precise cognitive training method, can make up for the deficiencies of patients in attention, memory, executive function, etc. through modular and step-by-step training mode. Its standardized training process and real-time feedback mechanism are linked to better training outcomes and higher patient participation [27, 28]. Evidence-based nursing provides comprehensive protection for cognitive correction therapy. By establishing a professional evidence-based nursing team, personalized nursing plans are formulated based on the latest scientific research evidence and the individual needs of patients [29]. Cognitive nursing treatment can complement computer-based cognitive correction therapy, further enhancing the effect of cognitive training; psychological nursing treatment can effectively alleviate patients’ anxiety and depression caused by illness and training stress, and improve patients’ treatment compliance; family support nursing creates a good rehabilitation environment, allowing patients to receive continuous support and guidance outside the hospital, consolidating the training effect; Some previous research results support the above statement [30, 31, 32]. In contrast, the conventional cognitive training used in the control group lacked pertinence and systematicity, the training content was relatively simple, and conventional nursing could not provide patients with personalized support, so the improvement effect was limited.

The results of this study show that, in terms of improving quality of life, the SQLS scores of all dimensions and the total score of both groups of patients decreased significantly after the treatment, and the decrease was greater in the observation group, indicating that evidence-based nursing combined with computer cognitive correction therapy can more effectively improve the quality of life of patients. The improvement of quality of life is comprehensively related to various factors such as cognitive function improvement, psychological state adjustment, and social function rehabilitation [33]. Improved cognitive function enables patients to better cope with various problems in daily life, improve their self-care ability and social skills [34]; while the psychological nursing, social function rehabilitation nursing and family support system in evidence-based nursing help patients alleviate negative emotions, improve social skills, enable them to better integrate into society, and reduce the loneliness and alienation caused by the disease; at the same time, it also reduces the psychological burden of patients and their families, allowing patients to feel more care and support, thereby improving the overall quality of life [35, 36, 37]. In addition, the combined treatment program may also improve the long-term quality of life of patients by improving their treatment compliance, reducing the risk of disease recurrence.

The limitations of this study are as follows: First, the retrospective study design may introduce selection and information bias; second, the 12-week treatment included both in-hospital and post-discharge phases, and long-term follow-up of patients was not conducted, making it impossible to clarify the long-term efficacy of the combined treatment; third, the sample size was limited to patients from our own hospital, which may restrict the generalizability of the research results. Future research could involve prospective, multicenter, large-sample studies with extended follow-up periods to further validate the long-term efficacy and safety of evidence-based nursing combined with computer-assisted cognitive correction therapy, providing more robust evidence for its widespread application in the rehabilitation of schizophrenia.

In conclusion, evidence-based nursing combined with computer-assisted cognitive correction therapy can effectively improve the cognitive function of patients with schizophrenia and significantly enhance their quality of life. It is a scientific and effective rehabilitation treatment program that deserves to be promoted and applied in clinical nursing in psychiatry.

## Conclusions

Patients receiving combined treatment of evidence-based nursing plus computer-assisted cognitive correction therapy tend to present preferable rehabilitation outcomes; this treatment is associated with improved cognitive function (including executive function, attention, information processing speed, and verbal fluency), significantly improve their quality of life, and has outstanding clinical application value. It can be regarded as one of the preferred options for rehabilitation treatment of patients with schizophrenia, and its clinical application is recommended.

## Availability of Data and Materials

The datasets used and analysed during the current study were available from the corresponding author on reasonable request.
